# Comparative retrospective review of perioperative analgesia using ultrasound-guided programmed intermittent erector spinae plane block for video-assisted thoracoscopic lobectomy

**DOI:** 10.1186/s12871-023-02338-z

**Published:** 2023-11-11

**Authors:** Xuefang Zhu, Wei Ye, Jinhong Chen, Jiwen Xiao, Weibing Zhao

**Affiliations:** Department of Anesthesiology, The Fifth People’s Hospital of Huan’an, No 1 Huaihe East Street, Huaiyin District, 223001 Huai’an City, Jiangsu Province China

**Keywords:** Erector spinae plane block, Perioperative analgesia, Programmed intermittent bolus, Continuous infusion, Video-assisted thoracoscopic lobectomy, Postoperative recovery quality

## Abstract

**Background:**

The retrospective cohort study was conducted to estimate the opioid-sparing anesthesia and limited side-effects with ultrasound (US)-guided ESPB using programmed intermittent bolus (PIB) or continuous infusion (CI) and standard opioid-based anesthesia in patients undergoing video-assisted thoracoscopic lobectomy (VATS).

**Methods:**

Patients underwent VATS were stratified into either control group or one of the two ESPB groups in a 1:2:2 ratio depending on whether PIB was implemented or not. The primary endpoint was intra- and post-operative opioids consumption over the first 48 h following surgery.

**Results:**

A total of 180 cases were included in the analysis. Cumulative perioperative opioid administration was found to be significantly different between PIB, CI and control group (both p < 0.001), and between PIB and CI group (p = 0.028). More specifically, the mean was 305.30 ± 51.35 mg, 339.68 ± 56.07 mg and 468.91 ± 79.84 mg in PIB, CI and control group. NRS scores at rest across all postoperative times were comparable in two ESPB groups, while significantly lower than control group, however, scores during exercising at postoperative 3, 6, 12 h were significantly lower in PIB group as compared to CI group. A wider anesthetized dermatomes with PIB was observed at 6, 24 and 48 h as opposed to the CI. The mean of levobupivacaine plasma concentration was significantly lower for PIB at postoperative 0.5, 12, 24 and 48 h after initiation than CI. However, local anesthetic toxicity was not observed in any of the two ESPB groups.

**Conclusions:**

When US-guided ESPB using PIB was performed preoperatively, it contributed to the minimization of intra- and post-operative opioid consumption due to better analgesia with a wider anesthetic dermatome opposed to conventional CI, whereas, it was also associated with lower risk of local anesthetic toxicity because of lower plasma concentration of levobupivacaine.

## Background

In the past decade, the video-assisted thoracoscopy surgery (VATS) was considered as a minimally invasive thoracic surgery which was associated with less trauma, less perioperative bleeding, better preservation of pulmonary function, earlier postoperative mobilization and better rehabilitation when compared to conventional thoracotomy. And it was consequently increased to almost half of thoracic surgery worldwide [1]. Nevertheless, there are some evidences that moderate to severe acute perioperative pain is still prevalent in VATS [2]. Therefore, effective intraoperative anesthesia and sufficient postoperative pain control remains a matter of concern for both anesthesiologists and thoracic surgeons [3]. Systemic opioids inhibit the sympathetic response and maintain hemodynamic stability, which is a powerful analgesia protocol for VATS. However, side effects including nausea and vomiting, respiratory depression, delirium and hyperalgesia delay recovery and prolong hospital stay [4]. Recently, a focus on multimodal and opioid-sparing analgesics has greatly expanded the options for acute pain management in minimally invasive thoracic surgery [5, 6]. Erector spinae plane block (ESPB) is one of the newest techniques proposed in 2016 for patients suffering from thoracic chronic pain. It deposits a local anesthetic (LA) deep into the erector spinae plane lying adjacent to transverse processes to provide regional anesthesia while avoiding the shortcomings of both opioids and neuraxial techniques [7]. Additionally, with the spread of LA to the thoracic paravertebral space thereby targeting the dorsal and ventral rami of spinal nerves, the ESPB may provide an alternative to the conventional thoracic paravertebral blocks with fewer risks of pneumothorax, which is recommended as a first-choice option for VATS [8, 9]. Here, we hypothesized that the ESPB using programmed intermittent bolus (PIB) under US guidance as a supplement to general anesthesia (GA) might mitigate the detrimental effects of opioid overuse, optimize pain control and avoid opioid-related complications for VATS. The study was designed to compare the perioperative analgesic efficacy and side-effects of US-guided ESPB using PIB or CI with the standard opioid-based anesthesia in patients undergoing VATS.

## Methods

### Study design and participants

**Fig. 1:**
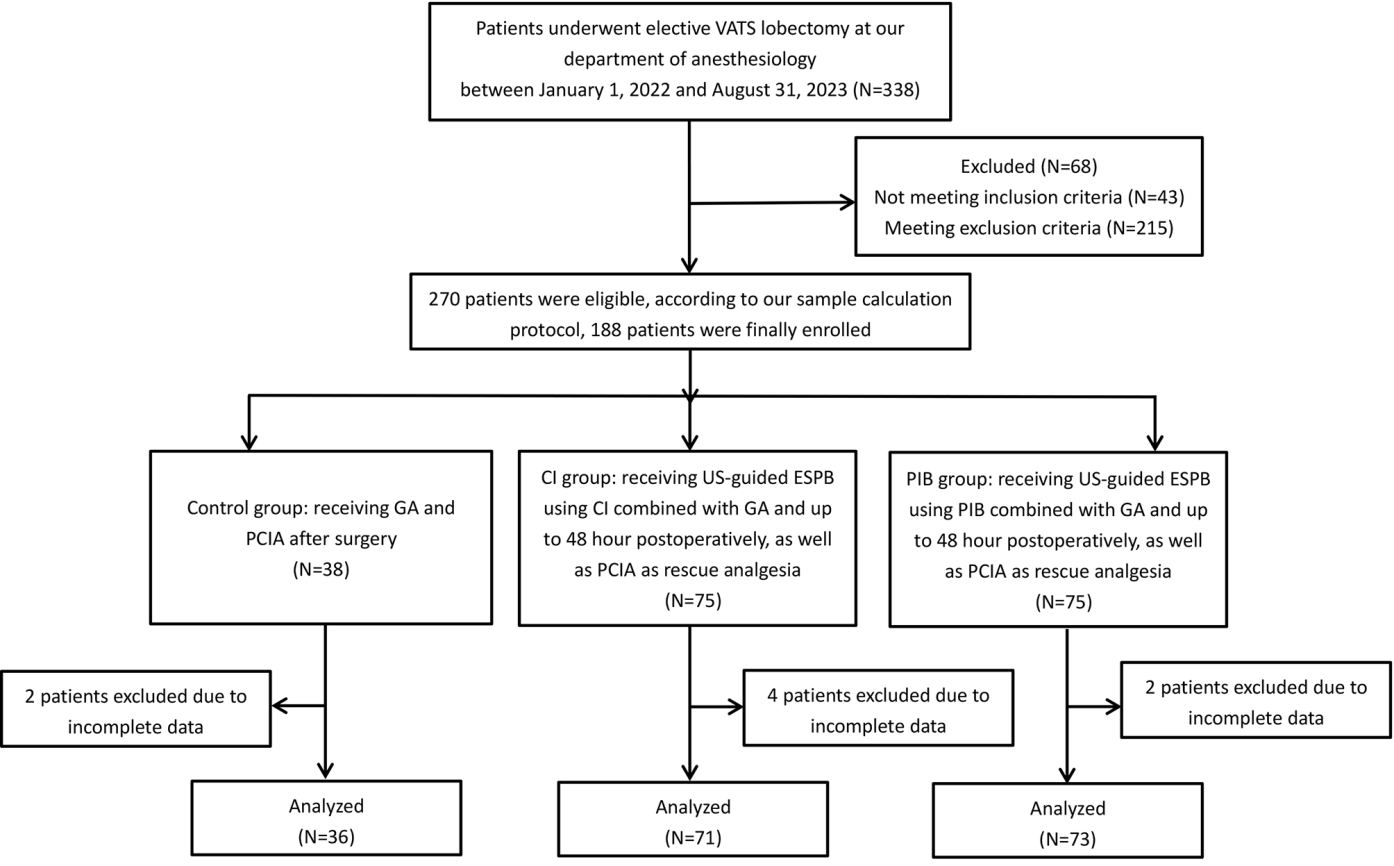
The diagram of patient recruitment

The study complied with the principles of the Declaration of Helsinki and the STROBE guidelines (Strengthening the Reporting of Observational Studies in Epidemiology) [10]. Ethical approval was obtained from Scientific Research Ethics Committee of the Fifth People’s Hospital of Huan’an (HAWY-KY-2021-177). Written informed consent was agreed to be waived because all data were inquired retrospectively from the electronic medical record system and postoperative follow-up. This retrospective cohort trial reviewed all patients who underwent elective VATS lobectomy at our anesthesiology department between January 1, 2022 and August 31, 2023. Inclusion criteria were as follow: (1) age between 20 and 80 years; (2) an American Society of Anesthesiologists (ASA) physical status classification of I-III. Exclusion criteria included: (1) age < 20 or > 80 years; (2) psychiatric disease; (3) coagulation disorders; (4) severe liver and/or renal dysfunction; (5) chronic opioid analgesics use (i.e., opioid prescribing for ≥ 90 days); (6) body mass index (BMI) ≥ 35 kg/m2; (7) unexpected conversion to open surgery; (8) shift and falling off of ESPB catheter; ⑼ violation of postoperative analgesia protocol including inability to use patient-controlled analgesia, receiving rescue analgesics other than dezocine and receiving other regional analgesia. According to our sample calculation protocol, a total of 188 patients were finally enrolled according to their admission time and divided into one of the following three groups in a 1:2:2 ratio based on their received perioperative anesthetic protocol. The control group, receiving GA and patient-controlled intravenous analgesia (PCIfsA) after surgery; the CI group, receiving US-guided ESPB with continuous infusion (CI) in conjunction with GA and up to 48 h postoperatively; the PIB group, receiving US-guided ESPB using a PIB regimen as an adjunct to GA and for a total of 48 postoperative hours (Fig. 1).

### Anesthesia protocol and surgery procedure

All surgeries were performed by the same surgical team strictly using the 3 ports technique located at the 4th, 6th and 7th intercostal space, a 40–100 mm length working window and 2 small ports for thoracoscopy and instruments.

Standard preoperative monitor including electrocardiogram (ECG), non-invasive blood pressure (NIBP), oxygen saturation (SpO2), invasive radial arterial blood pressure and bi-spectral index (BIS) were performed. And central venous catheter (CVC) into the subclavian vein was established under the real-time guidance of US [11]. Standard anesthetic protocol of GA was induced with propofol 1.5 mg/kg, sufentanil 0.5 ug/kg and cisatracurium 0.2 mg/kg. Anesthesia maintenance was performed with intravenous target-controlled infusion (TCI) of propofol 1.5-3.0 ug/ml and remifentanil 2.5–3.5 ng/ml to achieve a BIS value between 40 and 60, and intermittent infusion of cisatracurium as needed. When BIS＞60, propofol was given as a bolus at a dose of 0.5 mg/kg, and followed by an adjustment in the infusion rate until a BIS value in the range of 40–60 was maintained. Remifentanil was given intravenous bolus at a dose of 0.5 ug/kg, and then the infusion rate was increased in the range of 0.5 ug/kg/h to 1ug/kg/h for hypertension or tachycardia, If the infusion dose exceeded 1 mg/h, intravenous (IV) sufentanil 0.1 ug/kg was were used. On the contrary, the infusion rate was gradually reduced when hypotension or bradycardia was present. Additionally, fluids, atropine and norepinephrine were administered as needed when fluctuations in intraoperative mean arterial pressure (MAP) and heart rate (HR) of exceeded ≥ 20% from basal values. Vasoactive agents including atropine, ephedrine and norepinephrine were used when necessary. One-lung ventilation was performed and ventilator parameters were adjusted to maintain SpO_2_ at 95–100% and end-tidal carbon dioxide (EtCO_2_) at 35–45 mmHg. At the end of surgery, neostigmine 20 ug/kg and atropine 10 ug/kg were given to reverse residual muscle relaxation, as required.

### Ultrasound-guided erector spinae plane block procedure

**Fig. 2:**
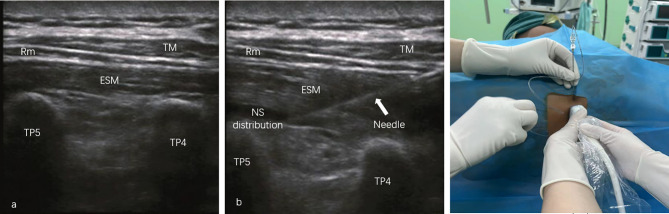
**(a)** The erector spinae muscle (ESM) was visualized superficial to the hyperechoic shadow of TP4 and TP5 and beneath the trapezius muscle (TM) and rhomboideus major (Rm) on the short axial view of US scan. **(b)** An epidural block cannula needle was inserted through the above-mentioned three muscles and advanced to the fascial plane between the TP and ESM using in-plane technique under the real-time guidance of US. Normal saline was administrated to dilate the fascia plane in order to confirm the correct position of the catheter in the targeted fascial space. **(c) **A epidural catheter was then inserted to the erector spinae plane through the cannula until the tip extended 5 cm beyond the tip of cannula according to the calibration. TP = transverse process; LA = local anesthetic

All blocks were performed by four consultant anesthesiologists who were experience in US-guided nerve block technique. Patients were placed in an ipsilateral lateral decubitus post induction of GA. A liner US transducer (5–12 Hz) was used to identify the 5th thoracic vertebral level by counting up from the 12th thoracic spinous process (SP). The transducer was then placed 3–4 cm lateral to the 5th thoracic SP in a longitudinal orientation in order to obtain the hyper-echoic acoustic line of the 5th thoracic transverse process (TP). The trapezius, rhomboid major and erector spinae muscles were subsequently recognized superficial to the hyperechoic TP shadow. After sterilization and local anesthesia, a 19-gauge epidural block cannula needle was inserted through the above-mentioned three muscles and advanced to the fascial plane between the TP and erector spinae muscle using in-plane technique under the real-time guidance of US (Fig. 2). After verification of needle tip precise position and a negative aspiration, 10 ml of normal saline was injected under real-time US guidance to dilate the fascia plane in order to confirm the correct position of the catheter in the targeted fascial space. Then, a 19-gauge epidural catheter was introduced to the erector spinae plane until the tip extended 5 cm beyond the tip of cannula according to the calibration, and secured with proper dressing after withdrawn of the cannula. Following catheterization, 20 ml of 0.25% levobupivacaine was gradually administrated, whilst visualizing cranial-caudal spread of the injectate by moving the transducer up and down. After the initiation bolus, the ESP catheter was connected to the electronic analgesia pump and subsequently commended with either the PIB and the CI regimen for intraoperative analgesia. The PIB patients received 0.125% levobupivacaine with programmed bolus of 20 ml 2 hourly as an adjunct to GA, while the CI patients received 0.125% levobupivacaine with CI at 10 ml/h rate in conjunction with GA, which was within the maximum volume and concentration of levobupivacaine suggested by previous evidences [12]. Although the infusion pressure and speed were different due to the differences of pulse quantity and frequency for drug administration between PIB and CI method, both two ESPB arms received the same total of 25 mg levobupivacaine every 2 h for analgesia of intraoperatively and a total of 48 postoperative hours.

### Postoperative pain management

In control group, patients received PCIfsA after extubation, where 0.2 ug/Kg of sufentanil was diluted in 0.9% normal saline to a total volume of 100 ml and delivered by a CI of 2 ml/h with 1 ml on-demand bolus dose and a 15-min lockout interval for a total of postoperative 48 h. As well as the two ESPB infusions, PCIfsA with the same concentration of sufentanil was initiated in two ESPB groups using the same pump parameters as the control group without background infusion after extubation. Bolus dose of 1ml with lockout interval 15 min was administrated for patient’s request analgesia and continued for 48 postoperative hours. 5 mg dezocine was intravenously injected as rescue analgesia if patients reported their NRS score at rest was greater than 3 before removing of patient-controlled analgesia (PCA) pump. Intravenous (IV) ondansetron 4 mg was used to treat postoperative nausea and/or vomiting (PONV), as needed.

### Outcome measures and data collection

Our primary outcome was cumulative opioid consumption including the amount of intraoperative TCI remifentanil and iv sufentanil, as well as sufentanil administered through PCIFSA over the first 48 h postoperatively. The units of opioid consumption were standardized to total intravenous morphine milligram equivalent (MME). Secondary outcomes were assessed as following: ⑴ the anesthetized dermatomes at 0.5, 6, 24 and 48 h after awaking form GA based on a pinprick test; ⑵ plasma concentration of levobupivacaine at 0.5, 3, 6, 12, 24 and 48 h after initiating the infusion for two ESPB groups; ⑶ The Chinese version of postoperative quality of recovery score at postoperative 48 h with the 15-item (QoR-15 C), which was validated as efficient and reliable as the original English version, comprises five subscales including pain (n = 2), physical comfort (n = 5), physical independence (n = 2), psychological support (n = 2) and emotional state (n = 4). Each item ranges from 0 to 10, therefore, the total score is scored from 0 to 150, and a higher score indicates better patient quality of recovery after surgery [13]. ⑷ postoperative pain severity during rest and while exercising (i.e., when coughing) evaluated by the 11-point Numerical Rating Scale (NRS) (0 = no pain to 10 = pain as bad as you can imagine) at 3, 6, 12 24 and 48 h after surgery. ⑸ the incidence of adverse events due to surgery or analgesia including pulmonary, pneumonia, respiratory dysfunction, hypotension, delirium, dizziness and PONV.

### Sample size calculation

Sample size calculation was performed using PASS version 16.0 software during analysis stage following data collection. Based on test involving 10 patients in each of the three groups and data about perioperative cumulative opioid consumption (expressed as IV MEQ) with 485.26 ± 62.41 mg without any regional anesthesia in our institution, we wanted to detect a shift in the mean of at least 30% reduction in ESPB groups. After consultation with the experts and review of the literatures, we set the mean of the control group to zero, the mean between continuous ESPB and programmed intermittent ESPB to 35.53 with a standard deviation (SD) of 0 to 80 by 5 based on the pre-trials. To achieve a 90% power at a Bonferroni adjusted significance level of 0.05/3 = 0.016 (two-tailed), the calculated number of patients in control group, CI group and PIB group were 30, 60 and 60, respectively using group sample size pattern of 1:2:2. Allowing for a possible dropout rate of 20%, we planned for 38, 75 and 75 patients in corresponding group.

### Statistical analysis

Statistical analysis was performed by SPSS software, version 22.0 (SPSS Inc, Chicago, IL, USA). Statistical significance was regarded as two-sided p value of < 5%. Kolmogorov-Smirnov test was used to exam the normality of data distribution. Data were described as mean ± SD, median ± inter-quartile range (IQR), frequency and percentage, respectively. Analysis of variance (ANOVA) and Kruskal-Wallis ANOVA were employed for evaluating group effects across three groups for means and medians. Post-hoc comparison was conducted at an adjusted significance level of 0.05/3 = 0.017. Fisher’s exact test was used for categorical variables. General linear mixed model (GLMM) was employed for repeated measures and the Student-Newman-Keuls multiple comparison post hot test was used to differentiate within groups because there were measurements from multiple time points.

## Results

A total of 338 patients were reviewed, after the exclusion of 68 patients, 188 patients were enrolled according to sample calculation protocol among 270 patients who were eligible (Fig. 1). 1, 4 and 2 cases in control, CI and PIB group were also excluded due to incomplete follow-up data, respectively. Therefore, data from 180 patients were included in the final analysis. Patients’ demographic characteristics were summarized and presented in Table 1, and profiles were comparable among three groups.

**Table 1:** Baseline characteristics of participating patients

Variables	Control group (N = 36)	CI group (N = 71)	PIB group (N = 73)	F/χ^2^ value	p
Age(years)	65.75 ± 8.62	64.90 ± 7.77	65.90 ± 7.49	0.091	0.913
Male sex, n (%)	23 (63.9%)	39 (54.9%)	43 (58.9%)	0.417	0.812
BMI	27.10 ± 2.84	26.81 ± 2.76	27.84 ± 3.16	0.664	0.518
ASA classification, n (%)				1.173	0.883
I	6 (16.7%)	14 (19.7%)	18 (24.7%)		
II	24 (66.7%)	46 (64.8%)	43 (58.9%)		
III	6 (16.7%)	11 (15.5%)	12 (16.4%)		
Smoking history, n (%)	7 (16.7%)	17 (23.9%)	16 (21.6%)	0.749	0.688
COPD, n (%)	4 (11.1%)	8 (11.3%)	10 (13.7%)	0.250	0.882
Hypertension, n (%)	8 (22.2%)	20 (28.2%)	17 (23.3%)	0.643	0.725
Diabetes, n (%)	7 (19.4%)	15 (21.1%)	18 (24.7%)	0.460	0.794
Coronary heart disease, n (%)	11 (30.6%)	22 (31.0%)	20 (27.4%)	0.250	0.883
TNM classification, n (%)				0.532	0.970
*I*	14 (38.9%)	30 (42.3%)	33 (45.2%)		
*II*	12 (33.3%)	22 (31.0%)	23 (31.5%)		
*III*	10 (27.8%)	19 (26.8%)	17 (23.2%)		
Intraoperative data					
*Propofol consumption (ug)*	1155.50 ± 128.41	1166.50 ± 141.62	1134.50 ± 186.53	0.222	0.801
*Intraoperative fluid blood loss (ml)*	2070.00 ± 290.37	2055.00 ± 337.91	1965.00 ± 313.34	0.652	0.525
*Surgery time (min)*	124.38 ± 25.95	119.88 ± 24.91	124.63 ± 28.66	0.203	0.817

BMI = body mass index; ASA = American Society of Anesthesiologists; COPD = chronic obstructive pulmonary disease; TNM = tumor node metastasis

### Perioperative opioids consumption

Table 2 demonstrated the cumulative opioid consumption during VATS and over the first postoperative 48 h. As hypothesized, there were significant difference in perioperative opioid consumption, the primary outcome, among three study groups with F = 37.348, p < 0.001 according to ANOVA. Post-hoc analysis revealed that both continuous and intermittent ESPB intervention showed significantly lower cumulative opioid consumption when compared to control group (339.68 ± 56.07 vs. 468.91 ± 79.84 mg, p < 0.001; 305.30 ± 51.35 vs. 468.91 ± 79.84 mg, p < 0.001). However, patients assigned to the PIB group had a significantly lower cumulative opioid consumption compared with patients assigned to the CI group with a mean difference of -34.39 (95%CI: -71.97, 3.20) (p = 0.028).

**Table 2:** Comparison of perioperative cumulative opioid consumption, duration of first-time usage of PCIA, number of PCIA press as rescue analgesia and QoR-15 scores among three groups within 48 h after surgery

Outcome		Control group (n = 36)	CI group (n = 71)	PIB group (n = 73)	F/χ^2^ value	P	Post-hoc analysis
**Perioperative cumulative opioid consumption** **(expressed as IV MEQ) (mg, mean ± SD)**		468.91 ± 79.84	339.68 ± 56.07	305.30 ± 51.35	37.348	< 0.001	PIB vs. control group: p < 0.001
							CI vs. control group: p < 0.001
							PIB vs. CI group: p = 0.028
**Duration of first-time usage of PCIA** **(min, median [IQR])**		35.52 ± 17.24	260.81 ± 63.89	312.80 ± 83.72	96.111	< 0.001	PIB vs. control group: p < 0.001
							CI vs. control group: p < 0.001
							PIB vs. CI group: p = 0.015
**Number of PCIA press** **(median [IQR])**		10 (8, 12)	4 (3, 5)	3 (2, 4)	26.40	< 0.001	PIB vs. control group: p < 0.001
							CI vs. control group: p < 0.001
							PIB vs. CI group: p = 0.031
**QoR-15 scores**	**At baseline**	143.02 ± 5.73	142.90 ± 5.25	143.20 ± 6.21	0.007	0.993	PIB vs. control group: p = 0.969
							CI vs. control group: p = 0.934
							PIB vs. CI group: p = 0.908
	**At postoperative 48 h**	73.30 ± 9.64	87.60 ± 13.28	91.80 ± 12.55	6.614	0.005	PIB vs. control group: p = 0.002
							CI vs. control group: p = 0.012
							PIB vs. CI group: p = 0.438

PCIA = patient controlled intravenous analgesia; IQR = interquartile range; SD = standard deviation; IV = intravenous; MME = morphine milligram equivalent; QoR = quality of recovery

### Postoperative NRS scores and QoR-15 scores

**Fig. 3:**
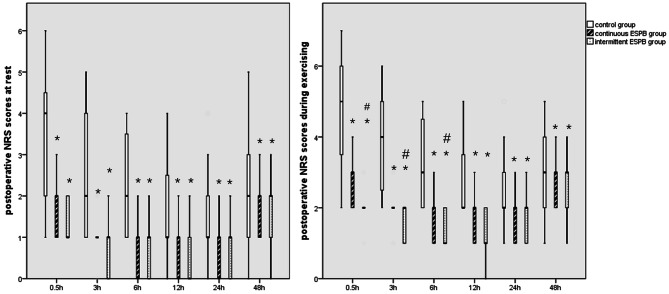
Box plot of NRS scores by study groups across different postoperative follow-up times. **(a)** postoperative NRS scores at rest; **(b)** postoperative NRS scores during exercising. *p < 0.05 versus control group; #p < 0.05 versus CI group. NRS = numerical rating scale, CI = continuous infusion

According to Kruskal-Wallis ANOVA and post-hoc comparison, lower postoperative NRS scores at rest at 3, 6, 12, 24 and 48 h were observed in both two ESPB groups in comparison with control group, respectively (adjusted p = 0.010 at 0.5 h, 0.001 at 3 h, 0.002 at 6 h, 0.002 at 12 h, < 0.001 at 24 h and 0.012 at 48 h versus control group in CI group; adjusted p < 0.001 at 0.5 h, < 0.001 at 3 h, < 0.001 at 6 h, < 0.001 at 12 h, 0.007 at 24 h and 0.031 at 48 h versus control group in PIB group). However, we found no significant differences, across different follow-up time points, between the two ESPB intervention groups with respect to median of NRS scores at rest (p = 0.172 at 0.5 h, 1.000 at 3 h, 1.000 at 6 h, 1.000 at 12 h, 0.731 at 24 h and 0.389 at 48 h). Moreover, compared with control group, NRS scores during exercising at postoperative 3, 6, 12, 24 and 48 h were all significantly lower in two ESPB groups (adjusted p = 0.006 at 0.5 h, 0.002 at 3 h, 0.006 at 6 h, 0.003 at 12 h, < 0.001 at 24 h and 0.025 at 48 h versus control group in CI group; adjusted p < 0.001 at 0.5 h, < 0.001 at 3 h, < 0.001 at 6 h, < 0.001 at 12 h, 0.007 at 24 h and 0.027 at 48 h versus control group in PIB group), while the median of NRS scores in PIB group were lower than those in CI group during exercising over the first 6 h (p < 0.001 at 0.5 h, 0.024 at 3 h, 0.018 at 6 h, 0.137 at 12 h, 0.731 at 24 h and 0.493 at 48 h) (Fig. 3). Compared with control group, total QoR-15 score was significantly increased while NRS pain score decreased in both two ESPB groups. In terms of data of different dimensions of QoR-15 scale, patients in PVB group had a better total scale (p < 0.001), physical comfort scale (p < 0.001) and less pain (p < 0.001). However, the other dimensions including psychological support, emotional state and activity ability between two groups did not reach statistically significant difference according to post-hoc analysis after ANOVA.

### Anesthetized dermatomes between two ESPB groups

**Fig. 4:**
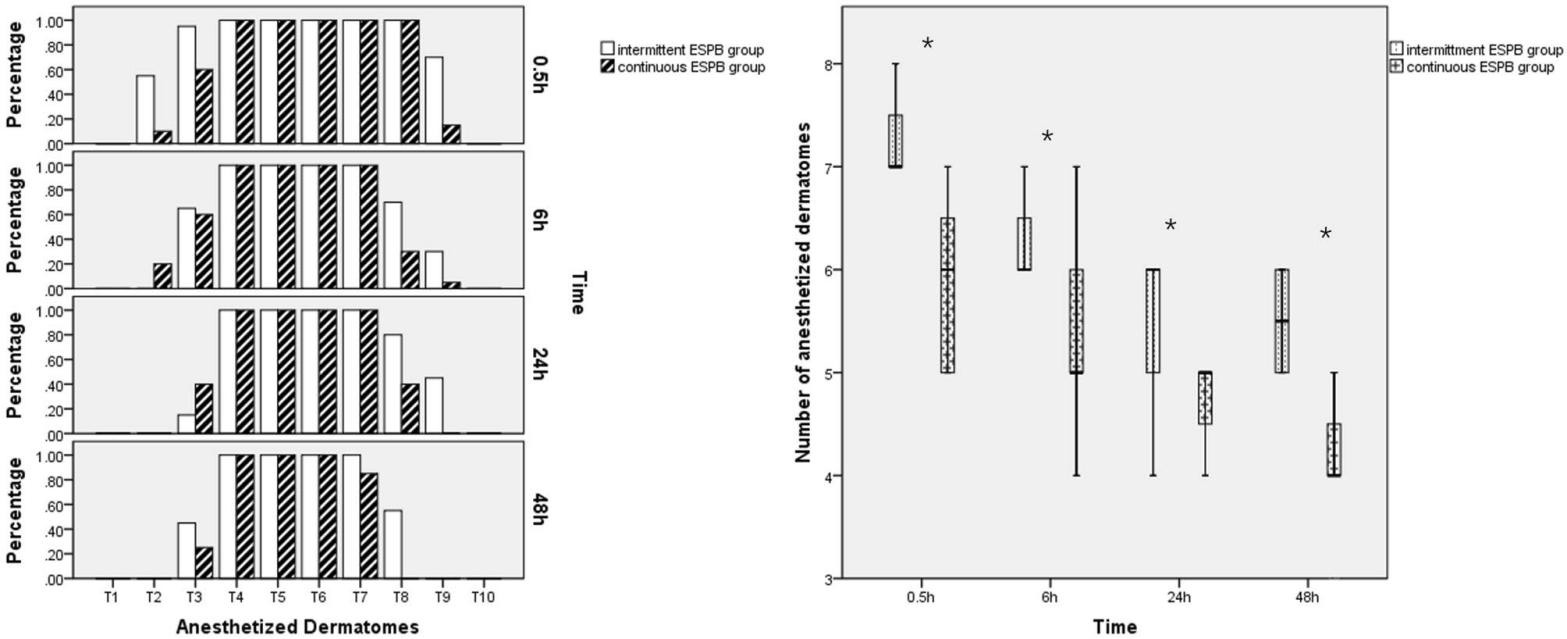
**(a)** Percentage of sensory loss at each thoracic dermatome observed at different postoperative follow-up time points in patients receiving continuous ESPB or intermittent ESPB, respectively. **(b) **Number of anesthetized dermatomes at different postoperative follow-up time points in both two ESPB groups. *p < 0.05, significantly wider anesthetized dermatomes were achieved in PIB group when compared with CI group. PIB = programmed intermittent bolus; CI = continuous infusion

The bar in Fig. 3 illustrated that percentage of anesthetized dermatomes on T2 was 55% and 10% in PIB and CI group at 0.5 h (p = 0.006), 95% vs. 60% on T3 (p = 0.020) and 70% vs. 15% on T10 (p = 0.001). At postoperative 6 h, percentage on T2, T3, T8 and T9 was 0% vs. 20% (p = 1.106), 65% vs. 60% (p = 1.000) 70% vs. 30% (p = 0.026) and 30% vs. 5% (p = 0.091), respectively. These percentages on T3, T8 and T9 at 24 h postoperatively were 15% vs. 40% (p = 0.155), 80% vs. 40% (p = 0.022), 45% vs. 0% (p = 0.001), respectively. At 48 h postoperatively, these percentage on T3, T7 and T8 were 45% vs. 25% (p = 0.320), 100% vs. 85% (p = 0.231) and 55% vs. 0% (p < 0.001). According to Mann-Whitney U test, patients who received intermittent ESPB had wider anesthetized dermatomes based on a pinprick test at 6, 24 and 48 h after awaking from GA as opposed to continuous ESPB (7 (IQR: 6.25, 7.75) vs. 6 (IQR: 4.25, 7.75), p < 0.001; 6 (IQR: 6, 6) vs. 5 (IQR: 4, 6), p = 0.018; 5.5 (4.5, 6.5) vs. 5 (4.25, 5.75), p = 0.038 and 5.5 (4.5, 6.5) vs. 4 (3.25, 4.75), p < 0.001) (Fig. 4).

### Plasma concentration of levobupivacaine between two ESPB groups

**Fig. 5:**
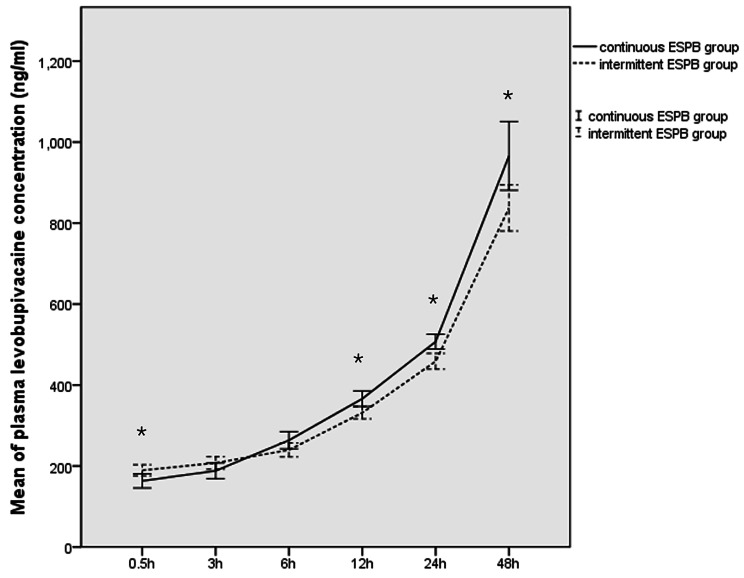
Means of the plasma concentration of levobupivacaine in patients receiving continuous ESPB or intermittent ESPB depending on time change during postoperative follow-up period, respectively. *p < 0.05 significant difference was observed between two ESPB groups

The mean of plasma concentration of levobupivacaine at 0.5, 3, 6, 12, 24 and 48 h after initiating the infusion were 163.17 ± 46.57, 188.28 ± 50.49, 263.72 ± 55.26, 366.28 ± 50.49, 507.20 ± 48.08 and 965.89 ± 223.09 ug/ml, respectively, in the CI group, versus 189.57 ± 37.41, 207.66 ± 42.22, 239.78 ± 46.83, 331.76 ± 41.42, 458.80 ± 53.46 and 837.40 ± 155.53 ug/ml, respectively in the PIB group. However, significant difference was observed at postoperative 0.5, 12, 24 and 48 h between two groups (p = 0.018 at 0.5 h, 0.111 at 3 h, 0.075 at 6 h, 0.005 at 12 h, 0.001 at 24 h and 0.012 at 48 h postoperatively, respectively) based on the Student-Newman-Keuls multiple comparison post hot test after GLMM for repeated measures (Fig. 5). No local anesthetic systemic toxicity events were observed in two ESPB groups.

### Postoperative adverse events

As the most common postoperative complication, significantly lower incidences of postoperative nausea and vomiting (PONV) were observed in two ESPB groups than in the control group up to 48 h postoperatively (26.3% vs. 10.7% vs. 8.0%, p = 0.017). A significantly decreasing trend of postoperative dizziness, pruritus and delirium was also observed in two ESPB groups than that in control group (21.1% vs. 8.0% vs. 6.7%, p = 0.042; 23.7% vs. 9.3% vs. 6.7%, p = 0.020 and 17.1% vs. 5.3% vs. 4.0, p = 0.032), however there was no differences between two ESPB groups. In contrast, no significant difference was observed in the incidence of postoperative pulmonary complications between two groups (2.6% vs. 1.3% vs. 0%, p = 0.418).

## Discussion

To the best of our knowledge, the trial is the first to evaluate the intermittent ESPB intervention on opioid-sparing perioperative analgesia taking the routine systemic analgesia and continuous ESPB as control in VATS. Our findings demonstrated that the PIB approach was associated with the lowest perioperative opioids consumption, lowest median of NRS scores during coughing over the first 6 h and fewest opioid-related complications after VATS, which suggested that the ESPB using PIB provided superior perioperative pain control coupled with decreased opioid requirement than continuous ESPB and standard opioid-based anesthesia.

In recent years, analgesic regimens for VATS vary significantly although it has been increasingly popular. The feasibility of opioid free anesthesia is proposed, because opioids are associated with high incidence of PONV, pruritus and respiratory depression when used solely for analgesia [14]. Currently, ROSPECT recommendations retrieving 1070 studies advised the addition of a regional analgesic technique as a component of multimodal analgesia (MMA) is strongly recommended as a first-choice option for VATS due to its efficacy on pain control and limited side-effects compared with TEA [8, 15].

Among the regional analgesic techniques, the ESPB is a novel inter-fascial plane block adopted in a variety of abdominal and thoracic surgery. The exact mechanism of this technique remains unknown. However, cadaver studies illustrated that the spread of local anesthetics was around the dorsal and ventral spinal nerves, the epidural space and even the sympathetic ganglion to provide appropriate combination of somatic and visceral analgesia [16]. Many previous studies were set out to systematically evaluated the qualitative nature of postoperative analgesic effects. US-guided ESPB using single injection was the most frequently used technique, as described, it is effective on postoperative pain management showing lower postoperative pain scores and accumulated opioid consumption when compared to a control group with no block [17–20]. Besides, it was affirmed as an inter-fascial plane technique easily to perform as the clear visualization of blockage target under US guidance leading to a lower risk of adverse events associated with neuraxial blocks [21]. However, there is limited duration of analgesic effect after single-shot ESPB ranging from 5 to 12 h using a 20 ml injection of 0.25-0.375% bupivacaine [17, 22]. Given that, the utilization of continuous ESPB via a catheter placement for prolonged analgesia has been proposed. Marco Cavaleri et al. firstly reported robotic-assisted thoracic surgery case series using continuous ESPB via a catheter positioned at the end of surgery as analgesic technique which provided effective postoperative analgesia longer than 48 h, thus showing a promising opioid-sparing effect [23]. In accordance with their results, a prospective randomized controlled study also illustrated the use of erector spinae catheter in VATS provided adequate analgesia following VATS as part of MMA and reduced opioid consumption and opioid-related side effects as compared to no intervention [24]. These available data suggested that continuous ESPB as a regimen of postoperative analgesia showed positive effect, which allowed to take a step further performing of continuous ESPB before surgery in order to get better intraoperative pain control and consequently reduce intraoperative opioid consumption. Subsequently, the first randomized controlled trial was conducted to compare the peri-operative analgesia provided by continuous ESPB block and TEA. Their findings showed the mean duration of analgesia was 20.60 ± 5.77 h and 21.72 ± 4.73 h in TEA and ESPB group, respectively. The intraoperative haemodynamics, postoperative rescue analgesia consumption in first 24 h, postoperative NRS scores at all follow-up time points were comparable in both the groups indicating that continuous ESPB was easy to perform, yet safe and effective alternative to TEA [25]. Consistent with the afore-mentioned evidence, the results of our study suggested that US-guide ESPB provided superior perioperative analgesia comparable to that of standard opioid-based balanced anesthesia as demonstrated by significantly lower administration of intraoperative TCI remifentanil, iv sufentanil and sufentanil through PCIfsA over the first 48 h following VATS in both continuous and PIB groups. Consequently, we found that the NRS scores at all follow-up time points were significantly lower in patients of two ESPB groups as compared with control group. Moreover, QoR-15 C scores of the patients regarding their postoperative recovery quality were significantly higher in patients of two ESPB groups compared to control group. Regarding the novelty of this study which lies in the performance of the promising ESPB to the MMA regimen preoperatively for patients undergoing VATS, we found that it could minimize not only postoperative but also intraoperative opioid consumption. It decreased perioperative opioid need, improved postoperative pain management and reduce opioid-related adverse events in accordance with previous trial [26].

The other area of interest concerning the use of PIB administration, the consensus among experts is that PIB of local anesthetics achieved a better spread and analgesia as compared to continuous peripheral nerve blocks as compared to CI [27]. Recently, many studies employed the efficacy and safety of US-guided ESPB using PIB when compared with alternative analgesia method including standard opioid anesthesia without any regional anesthesia, thoracic paravertebral block (TPVB), intrathecal morphine (ITM) and TEA, which confirmed that intermittent ESPB successfully decreased postoperative opioid consumption and demonstrated respectively better or non-inferior early postoperative analgesia as compared with no intervention or TPVB, ITM and TEA. Additionally, the intermittent technique even had a better side effect profile than neuraxial blocks [15, 28–30]. Based on the encouraging results of previous studies as mentioned above, our results also confirmed our hypothesis that intermittent ESPB analgesia reduced perioperative opioid consumption and provided significantly better postoperative analgesia with fewer incidence of postoperative side effects related to opioid overuse as compare to the control group, which could diminish postoperative organ dysfunction while facilitating early mobility and postoperative recovery in accordance with Enhanced Recovery after Surgery (ERAS) Protocols [31]. To the best of our knowledge, there has been only two randomized studies compared the efficacy of PIB and CI for ESPB in VATS. Only one of them drew the conclusion that PIB resulted in a larger anesthetized area and required a lower anesthetic dose to maintain the analgesic effect [32, 33]. Cadaveric evidence described that an ESPB with 20 ml of injectate provided neural foraminal and epidural spread across 2 to 5 vertebral levels in magnetic resonance imaging [34]. And the published trials described the application of ESPB with 20 ml of CI at 8 ml/h at the 5th thoracic transverse process for VATS allowed cephalocaudal spread covering 5 to 9 dermatomes which provided a reasonable analgesic efficacy [30, 35]. According to our results, the continuous ESPB resulted in the median of discernable cutaneous sensory loss to pinprick covering 4 to 6 dermatomes at all time points assessment. However, the intermittent ESPB showed a significantly wider sensory block of with the median of 5.5 to 7 anesthetized dermatomes during follow-up period. Therefore, we could explain that maintenance of ESPB analgesia using PIB model resulted in a lower consumption of opioid with better perioperative analgesia than continuous infusion in our results, because it could provide LA at planned intervals with a higher injection pressure to increase the extent of anesthesia and prevent the range of anesthetized dermatomes becoming gradually narrower if the LA is administrated through the boluses at fixed, scheduled time intervals [36]. Theoretically, the plasma concentration of LA could be stabilized when the LA is administrated at a constant rate according to pharmacokinetic parameters, whereas, the plasma concentration would show as a serrated increase pattern if the PIB model was used. This resulted in a significantly lower plasma concentration of levobupivacaine in PIB group at the 0.5, 12, 24 and 48 h time point as compared to CI, which might result in a lower risk of local anesthetic toxicity reported as the most common adverse events after ESPB [37]. And consequently enhanced postoperative patients’ recovery was achieved in our results due to its effective analgesia and safety. Therefore, this simple novel method might also be feasible even on patients undergoing thoracic surgery while on extracorporeal membrane oxygenation (ECMO) for providing circulatory and respiratory support [38].

There were some limitations: Firstly, the present study was carried out in the single center, and follow-up was only conducted within 48 h after VATS, longer observation in multiple centers should be performed in a well-designed randomized trial in the future. Secondly, VAS scores which was adopted to evaluate postoperative pain intensity was highly subjective. Thirdly, the present study only report one model of PIB, future studies would be required to identify the best factors for reliable ESPB using PIB including the programmed bolus volume, time interval between doses and concentration of local anesthetic for VATS. Fourthly, we did not compare intermittent ESPB with neuraxial blocks, therefore, a well-designed randomized study was required in future.

In conclusion, when US-guided ESPB using PIB was performed preoperatively, it contributed to the minimization of intra- and post-operative opioid consumption when compared with continuous ESPB and standard opioid-based anesthesia. It provided superior postoperative analgesia, larger anesthetized dermatomes, lower risk of local anesthetic toxicity and fewer incidence of postoperative side effects related to opioid overuse as opposed to continuous ESPB, which might be a viable analgesic regimen for VATS.

## Data Availability

The data that support the findings of this study are available from the corresponding author, Professor Weibing Zhao, upon reasonable request.

## Abbreviations

ESPB: erector spinae plane block
VATS: video-assisted thoracoscopy surgery
GA: general anesthesia
CI: CI
PIB: programmed intermittent bolus
MME: morphine milligram equivalent
PCIFSA: patient-controlled intravenous analgesia
VAS: Visual Analogue Scale
QoR: quality of recovery
PONV: nausea and vomiting
TEA: thoracic epidural analgesia
ITM: intrathecal morphine
TPVB: thoracic paravertebral block
MMA: multimodal analgesia
US: ultrasound
ASA: American Society of Anesthesiologists
BMI: body mass index
NS: normal saline
ECG: electrocardiogram
NIBP: non-invasive blood pressure
SpO2: oxygen saturation
BIS: bi-spectral index
TCI: target-controlled infusion
MAP: mean arterial pressure
HR: heart rate
EtCO2: end-tidal carbon dioxide
ICU: intensive care unit
CSF: cerebrospinal fluid
GLM: general linear model
ANOVA: analysis of variance
SD: standard deviation
IQR: inter-quartile range
